# Evaluation of Commercially Available Products of *Cannabis sativa* L. Inflorescences to Identify Their Contents of Elemental and Phenolic Compounds

**DOI:** 10.3390/foods14071208

**Published:** 2025-03-29

**Authors:** Katarzyna Wozniczka, Agnieszka Viapiana, Anna Roszkowska, Alina Plenis, Tomasz Baczek, Pawel Konieczynski

**Affiliations:** 1 Department of Pharmaceutical Chemistry, Faculty of Pharmacy, Medical University of Gdansk, Al. Gen. J. Hallera 107, 80-416 Gdansk, Poland; katarzyna.wozniczka@gumed.edu.pl (K.W.); anna.roszkowska@gumed.edu.pl (A.R.); tomasz.baczek@gumed.edu.pl (T.B.); 2 Department of Analytical Chemistry, Faculty of Pharmacy, Medical University of Gdansk, Al. J. Hallera 107, 80-416 Gdansk, Poland; agnieszka.viapiana@gumed.edu.pl (A.V.); alina.plenis@gumed.edu.pl (A.P.)

**Keywords:** hemp tea, elements, phenolic compounds, antioxidant activity, statistical methods

## Abstract

Evaluation of 12 commercially available products of *Cannabis sativa* L. was performed to find similarities and differences in their composition. The contents of metallic elements determined by flame atomic absorption spectrometry (FAAS) made it possible to order microelements as follows: Fe > Mn > Zn > Cu. As for the contents of macroelements, the pattern was Ca > K > Mg > Na. Analyses of hemp samples were also performed via assays of their phenolic compounds and ascorbic acid by UV/Vis spectrophotometry. The antioxidant activity was determined based on the Ferric-Reducing Antioxidant Power (FRAP), Cupric-Reducing Antioxidant Capacity (CUPRAC), 2,2-diphenyl-1-picryl-hydrazyl-hydrate (DPPH), and ABTS Radical-Scavenging Activity. We concluded from the application of cluster analysis (CA) and principal component analysis (PCA) that several hemp samples (for example, the sample from Italy) were well-separated from the others due to their different chemical composition. In conclusion, the results achieved with the statistical methods are helpful in interpreting the results obtained for hemp samples and reveal characteristic tendencies among the investigated samples due to their contents of metals, phenolic compounds, ascorbic acid concentrations, and antioxidant properties.

## 1. Introduction

*Cannabis sativa* L. has been cultivated since ancient times as a fiber plant, as well as for its pharmacological and mood-enhancing properties [1]. The pharmacological properties of the Cannabis plant are usually associated with phytocannabinoids, such as Δ-9-tetrahydrocannabinol (Δ9-THC), cannabidiol (CBD), and cannabigerol (CBG) [2]. Besides phytocannabinoids, *Cannabis sativa* contains hundreds of other compounds such as terpenoids and non-cannabinoid phenolic compounds [2,3]. The pharmaceutical materials are *Cannabis inflorescences* (Cannabis flos), but the richness of various bioactive or nutritive compounds allows for using all morphological Cannabis plant parts in various applications [4]. Moreover, the composition of compounds specific to the Cannabis plant gives it unique properties, including significant antioxidant activity. Antioxidant properties have been described for *Cannabis inflorescences* [5,6,7], leaves [8,9,10], seeds [11,12,13], and roots [14]. Potent antioxidant activity can be determined by studying the phytocannabinoid antioxidant activity itself [15] and the contents of polyphenolic compounds (i.e., flavonoids and phenolic acids) [8,12]. According to the work of Jin et al. [4], among the plant parts, the richest in flavonoids are the leaves and then the inflorescences of the Cannabis plant. Moreover, inflorescenses’ polyphenol content and antioxidant activity correlate with the harvest time and maturation of the flowers [5]. Moreover, the phenolic composition and antioxidant activity can differ between cultivars [5].

Unique flavonoid characteristics of Cannabis are produced by cannaflavins A and B, whose anti-inflammatory properties were proven in in vitro and in vivo studies, revealing that they are thirty times more potent as anti-inflammatories than aspirin [16,17]. Cannabis seeds’ antioxidant activities can be associated with specific lignanamides (i.e., cannabisines) [12].

*Cannabis sativa* has a deep root system, therefore absorbing elements from the soil, and can be used in soil recultivation [18,19]. Cannabis plants can accumulate heavy metals from the environment and also offer a potential source of essential microelements [20]. Because of the increasing popularity of Cannabis products, the aim of this study was to analyze the composition of phenolic compounds, ascorbic acid content, and elemental concentration in commercially available samples of Cannabis intended for human consumption.

## 2. Materials and Methods

### 2.1. Standards and Reagents

Gallic acid (GEA), quercetin (QE), ascorbic acid (AA), caffeic acid (CA), p-coumaric acid (pCA), vanillic acid (VA), ferulic acid (FA), cinnamic acid (CNA), protocatechuic acid (PCAT), rutin (RUT), apigenin (API), 2,2-diphenyl-1-picrylhydrazyl (DPPH reagent), 2,2-azinobis (3-ethylbenzothiazoline-6-sulfonic acid), diammonium salt (ABTS reagent), 4-chloro-7-nitrobenzofurazan (NBD-Cl), ammonium acetate, and neocuproine were purchased from Sigma-Aldrich (St. Louis, MO, USA). Aluminum chloride (AlCl_3_) was obtained from Across Organics (Morris Plains, NJ, USA), and high-performance liquid chromatography (HPLC)-grade methanol (MeOH) was obtained from Avantor (Central Valley, PA, USA). The other reagents were obtained from POCh (Gliwice, Poland). The redistilled water was prepared by triple distillation of water in a Destmat^®^ Bi-18 system (Heraeus Quarzglas, Hanau, Germany).

### 2.2. Plant Material and Extraction Procedure

Twelve samples of *Cannabis sativa* were analyzed. Eight of them sold as “hemp tea” were obtained from herbal shops, Konopna Farmacja and Zielarnia Warmińska, located in Olsztyn, Poland, as well as web stores (konopiezmazur.pl, konopienamaksa.pl), mostly with no specified origin or cultivar. Four of them (nos. 8–12) were kindly provided by Kombinat Konopny S.A. All samples were pulverized in a cooled mill Knifetec 1095 grinder (Foss Tecator, Höganäs, Sweden). They are described in Table 1.

For the infusions, 2 g of plant material was mixed with 50 mL of boiling redistilled water and left to rest for 15 min. The extracts were then filtered through Whatman No. 4 paper. To obtain hydromethanolic extracts, 0.5 g of each sample was sonicated with 3 mL of methanol–water mixture (80:20, *v*/*v*) for 15 min at 25 °C using an ultrasonic bath (Emag, Salach, Germany). Then, the suspension was centrifuged in an EBA-20S centrifuge (Hettich, Tuttlingen, Germany) for 5 min at 8000 rpm, and the supernatant was transferred into a volumetric flask. This procedure was repeated twice, and the obtained extracts were combined and diluted up to 10 mL with a mixture of methanol–water (80:20; *v*/*v*) according to procedures developed before [21]. For element content analysis, the sample preparation protocol was described elsewhere [22].

### 2.3. Analysis of Phenolic Compounds, Total Phenolics, and Other Analytes

Phenolic compounds were determined using UV/Vis spectrophotometry as previously described by Polumackanycz et al. [21]. The total phenolic (TPC), flavonoid (TFC), phenolic acid (TPAC), and ascorbic acid (AA) contents of the hemp extracts were determined using the methods also described in the cited paper.

### 2.4. Antioxidant Activity

Antioxidant activity was assayed by the DPPH Scavenging Activity Method [23], a CUPRAC assay according to the method of Apak et al. [24], and according to the ABTS Radical-Scavenging Activity determined by the method of Arnao et al. [25]. The original methods’ modifications were previously described by Polumackanycz et al. [21].

### 2.5. Statistical Analysis

For hemp samples, all assays were carried out in triplicate. The results are expressed as mean values and standard deviations. Variations between samples were evaluated using one-way analysis of variance. The relationship between the phenolic composition and antioxidant activity was analyzed by Pearson correlation analysis, and unsupervised pattern recognition analysis using principal component analysis (PCA) was applied. Statistical analysis was performed using Statistica 10 software (StatSoft Inc., Tulsa, OK, USA).

## 3. Results and Discussion

### 3.1. Elements

Results of metallic elements’ determination by the FAAS technique are presented in Table 2, where values followed by the same letter for element do not differ significantly (*p* < 0.05). The microelements were determined to have the following order: Fe > Mn > Zn > Cu. As for the contents of macroelements, the pattern was Ca > K > Mg > Na. Through a post hoc Tukey test, the statistically significant differences were determined, as indicated by the letters in Table 2.

In several samples, a high level of the investigated elements was assayed. For example, a high concentration of iron was determined in sample 11, originating from Italy, where Fe was above 937 mg/kg of dry weight (DW). This amount of iron was much higher than in the other analyzed samples of Cannabis, where the Fe level was in the range between 100 and 200 mg/kg DW; however, in sample 6, it was found to be 723 mg/kg DW. Manganese was found to be the highest in sample 10, at above 200 mg/kg DW. In the other samples of Cannabis, the Mn level was determined in lower amounts, from about 36 to 170 mg/kg DW. Another microelement, Zn, was found to be the highest in sample 5, at almost 100 mg/kg DW, but in other samples, Zn was determined as being from 25 to 85 mg/kg DW. The copper level was quite varied in the studied Cannabis samples. In some of them (samples 8, 9, and 10), it was found to be as high as 22–33 mg/kg DW. In other samples, the Cu level was much lower, from 1.20 mg/kg DW in sample 11 to almost 8 mg/kg DW in sample 8.

Similar analysis of the results for macroelements was conducted. Magnesium was found to be the highest in sample 7, where the concentration was 8.8 mg/g dm, and the lowest in samples 2 and 3, at about 5.4 mg/g dm. Calcium was also found to be the highest in sample 7 from the firm “Kombinat konopny”, at about 75 mg/g dm, and on the opposite side, the lowest Ca level was in sample 3, at above 30 mg/g dm. Potassium was found at a high level in sample 12, also from “Kombinat konopny”, at 39 mg/g dm, and the lowest K concentration was noted in sample 9, at 3.7 mg/g dm. On the other hand, sodium was the macroelement with the lowest range of concentrations, from about 10 mg/kg dm in the Cannabis sample from Italy (sample 11) to about 340 mg/kg dm in sample 7 from the firm “Kombinat konopny”.

Our results are concurrent with other studies focused on the analysis of elements in Cannabis plants, as well as those on the levels of micro- and macroelements in hemp [26,27,28]. When analyzing the concentrations of particular elements in hemp samples by comparing the data from the literature with our results, several differences were noted. For example, Alonso-Esteban et al. [28] analyzed nine elements in eight hemp cultivars. The iron levels determined in our samples were significantly higher than those assayed in whole and hulled hemp seeds.

### 3.2. Phenolic Composition

The results of phenolic profiling and investigation of the ascorbic acid content are presented in Table 3. The values followed by the same letter for each parameter for each extract do not differ significantly (*p* < 0.05). For all presented parameters, the highest values were obtained for aqueous solutions. This corresponds with the study by Benkirane et al. [12], where for aqueous extracts of hemp seeds, TPC values were higher than for hydromethanolics. Moreover, the obtained results for the TPC assay correspond with those in the study of André et al. [5] on hemp hydromethanolic extracts (70:30, *v*/*v*), where TPC ranged from 4.72 to 22.05 mg GEA/g and differed between cultivars and maturation stages. Higher values for aqueous and methanolic extracts derived from Cannabis leaves were described by Ahmed et al. [9] (29.98 and 36.42 mg GEA/g, respectively). In contrast to our study, in the cited work of Ahmed et al. [9], there was no TF content in aqueous solutions, but much higher values were detected for pure methanolic extracts (59.03 mg QE/g). To the best to our knowledge, there has been no study analyzing the TPA content in the green parts of the Cannabis plant, but the data obtained in this study are higher than the results obtained by Alonso Esteban et al. [11] from hydromethanolic extracts of hemp seeds (0.266–1.20 mg/g extract). In the analyzed samples, the AA content was much higher than in Cannabis seeds, as determined by Bhatt et al. [29]. The reasons for the differentiation of data in the cited literature may be different results of the extraction procedure, different Cannabis cultivars analyzed, and also variability in morphological plant parts.

### 3.3. Antioxidant Activities

In this study, the highest antioxidant values were achieved for water–methanol extracts in the CUPRAC, ABTS, and DPPH methods, while in the results obtained by the FRAP method, higher values were achieved for aqueous extracts (Table 4). This finding contradicts data published by Aazza [30], where increasing the percentage of methanol in the solution used for extraction increased the antioxidant capacity of compounds analyzed by the FRAP method. Moreover, the results obtained differ from those published by Cásedas et al. [31], where 17.75 and 21.94 µmol Fe^2+^/g values were obtained for aqueous and hexane extracts, respectively. In a recently published study [32] that examined the antioxidant properties of hemp leaf extracts, the values for the DPPH method are similar (in the range of 2.20–10.29 mg TE/g DW), but the results for the ABTS method are much lower (7.20–10.37 mg TE/g DW). This may be due to the authors’ use of organic solvents without an aqueous component and a different extraction method; in addition, the samples used in this study also included inflorescences, which, given their contents of phytocannabinoids and terpenes, may have higher antioxidant properties than leaves [32].

**Figure 1:**
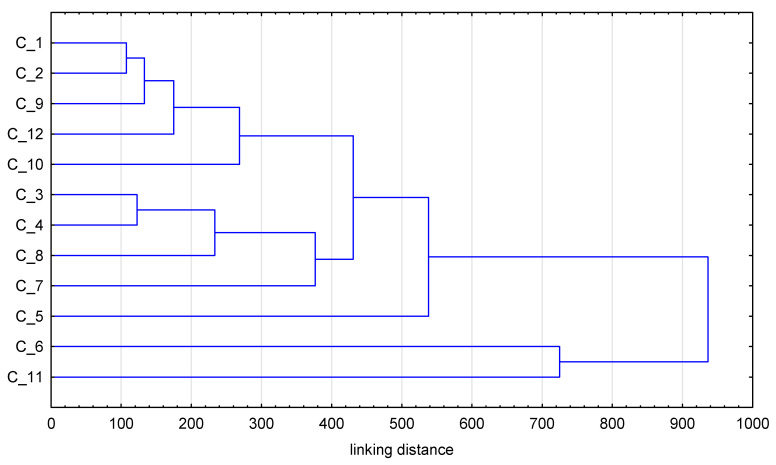
Cluster analysis results for 12 investigated hemp samples.

**Figure 2:**
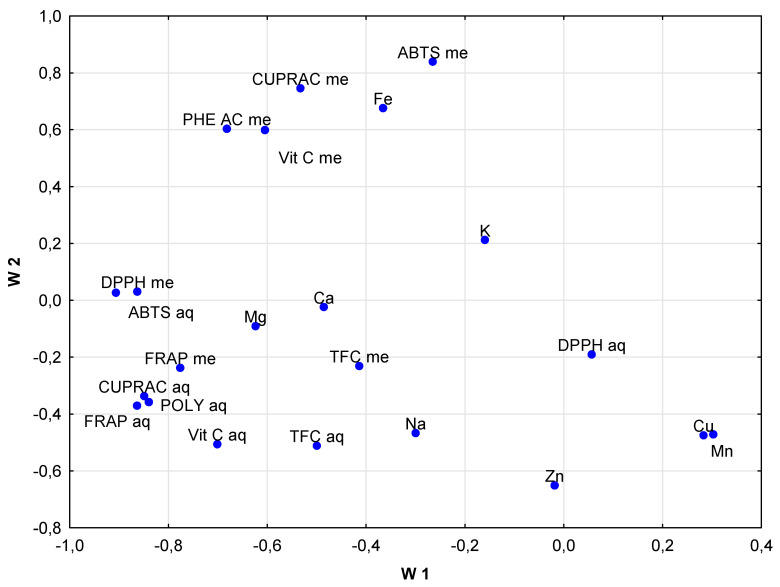
Loading plot obtained for PCA results for the studied 12 hemp samples.

**Figure 3:**
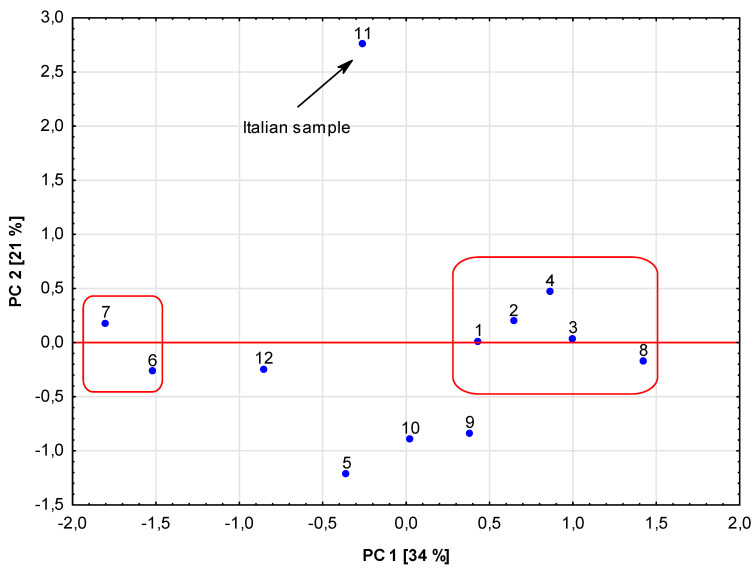
Score plot for the first two principal component analyses for the analyzed 12 hemp samples.

In order to obtain a more complex interpretation of the experimental results, statistical methods—a cluster analysis, CA and principal component analysis, PCA—were applied. The cluster analysis results presented in Figure 1 show that some of the studied samples were characterized by similar metallic element contents, and this was the reason for their grouping in close or distant clusters. Such a situation can be noted for samples 1 and 2, representing mixtures of morphological parts of hemp, regardless of the fact that the analyzed hemp samples originated from two different plantations. A similar remark can be made for samples 3 and 4, where the plantations were different, too. On the other hand, it is quite clear, for example, that sample 11 and sample 6 have significantly different levels of the studied metals; thus, they can be found at a relatively long distance from the other analyzed hemp samples, as is visible in the dendrogram (Figure 1). It was found via PCA that elements such as Cu and Mn, as well as the antioxidant activity determined by the DPPH, FRAP and CUPRAC methods, had the greatest influence on clustering (Figure 2). That is perhaps justified by the fact that these metals can be electroactive and their species can influence the antioxidant properties of the secondary metabolites present in the plant samples. PCA is a method that also allows us to present the distribution of the analyzed samples in clear plots, showing the locations of samples in two-dimensional space for PC1 vs. PC2 (Figure 3). It can be concluded from this plot that the distribution of the studied samples is somehow similar to the one obtained by the CA method. Again, sample 11 originating from Italy is well-separated from the others due to its different chemical composition. On the other hand, in contrast to the CA results, clustering of samples 6 and 7 caused by their similar levels of analytes, as well as of the samples with numbers 1–4 and 8, can be seen. In the case of samples 6 and 7, they originated from different plantations, but both of them contained inflorescences, which can justify their common location in Figure 3 but different clustering in Figure 1.

To conclude, the results of the statistical methods, namely CA and PCA, are helpful in interpreting the results obtained for hemp samples and show characteristic tendencies among the investigated samples due to their contents of metals and antioxidant properties.

## 4. Conclusions

The combination of a determination of elements together with an analysis of phenolic compound contents and antioxidant activity can offer a valuable tool for detecting similarities and differences among analyzed hemp samples. Moreover, the interpretation of experimental results produced with the use of statistical tools, such as cluster analysis and principal component analysis, could become a standard practice in the recognition of the origins of hemp samples available in the market. However, the statistical methods mentioned above are not the only ways to interpret the results of hemp analyses, and in the future, studies should be extended to include a greater number of Cannabis inflorescences originating from inside and also outside of Europe, to develop a larger research base supporting the interpretation of the obtained results.

## Figures and Tables

**Table 1:** Characteristics of *Cannabis sativa* samples used in this study.

No	Sample name on the Package	Morphological Part	Origin	Cultivar
1	Konopie z Mazur Hemp tea	Inflorescence 80%/leaves 10%/seeds 10%	Poland	*Santica*
2	Koyi Hemp tea	Leaves 50%/inflorescence 50%	unknown	unknown
3	Carun Young hemp leaves	Leaves 100%	Czech Republic	unknown
4	Konopie na Maksa Hemp tea	Leaves 50%/inflorescence 50%	unknown	unknown
5	Marysieńka Hemp tea	Leaves 25%/inflorescence 65% and seeds 10%	Poland	unknown
6	Konopie Hemp tea	Inflorescence 50%/leaves 50%	unknown	unknown
7	Herbatka Konopna Hemp tea	Inflorescence 100%	unknown	unknown
8	Herbatka Konopna Hemp tea	Inflorescence 100%	Poland	Finola
9	*Cannabis sativa*	Aerial parts 100%	Lithuania	Futura
10	*Cannabis sativa*	Aerial parts 100%	Poland	Fibror
11	*Cannabis sativa*	Aerial parts 100%	Italy	Enctalina
12	*Cannabis sativa*	Aerial parts 100%	Poland	Felina

**Table 2:** Results of elements’ determination. The arithmetic mean ± SD is shown in mg/kg DW for Fe, Mn, Zn, Cu, and Na and in mg/g DW for Mg, Ca, and K.

No.	Fe	Mn	Zn	Cu	Na	Mg	Ca	K
1	202.15 ± 2.61 ^e^	54.25 ± 0.18 ^d^	44.42 ± 0.25 ^e^	2.60 ± 0.07 ^ac^	135.0 ± 0.30 ^i^	5.6 ± 0.05 ^b^	54.2 ± 0.50 ^a^	29.9 ± 0.20 ^k^
2	172.84 ± 0.78 ^a^	88.24 ± 0.14 ^g^	39.57 ± 0.07 ^b^	4.55 ± 0.07 ^abc^	76.0 ± 0.90 ^e^	5.4 ± 0.04 ^c^	49.5 ± 0.51 ^g^	16.0 ± 0.14 ^g^
3	190.35 ± 1.13 ^d^	106.45 ± 0.18 ^h^	54.51 ± 0.25 ^c^	4.11 ± 0.07 ^abc^	113.3 ± 1.50 ^g^	5.4 ± 0.04 ^ac^	30.7 ± 0.33 ^e^	9.3 ± 0.08 ^c^
4	190.35 ± 2.45 ^a^	36.75 ± 0.24 ^a^	43.85 ± 0.40 ^b^	7.78 ± 0.12 ^b^	41.0 ± 0.10 ^c^	6.4 ± 0.05 ^d^	58.9 ± 0.55 ^cd^	16.4 ± 0.13 ^h^
5	176.87 ± 2.53 ^a^	167.15 ± 0.18 ^k^	98.84 ± 0.18 ^i^	5.72 ± 0.37 ^ab^	221.0 ± 0.50 ^a^	6.6 ± 0.06 ^ef^	71.1± 0.62 ^h^	18.1 ± 0.16 ^i^
6	723.03 ± 1.45 ^b^	55.32 ± 0.19 ^c^	55.32 ± 0.19 ^g^	7.13 ± 0.07 ^ab^	130.5 ± 0.50 ^h^	5.5 ± 0.05 ^ab^	36.3 ± 0.32 ^b^	10.7 ± 0.05 ^d^
7	180.34 ± 0.26 ^a^	37.57 ± 0.32 ^b^	25.52 ± 0.14 ^d^	4.86 ± 0.07 ^abc^	343.5 ± 0.40 ^k^	8.8 ± 0.07 ^i^	74.9± 0.67 ^i^	14.4 ± 0.10 ^e^
8	122.44 ± 1.30 ^c^	136.78 ± 0.21 ^j^	28.83 ± 0.07 ^a^	22.68 ± 0.07 ^d^	230.2 ± 0.20 ^j^	5.5 ± 0.04 ^ab^	37.2 ± 0.32 ^b^	15.0 ± 0.11 ^g^
9	138.19 ± 1.91 ^b^	128.48 ± 0.18 ^i^	84.51 ± 0.29 ^h^	22.29 ± 0.46 ^d^	87.6 ± 0.50 ^f^	6.7 ± 0.05 ^f^	44.0 ± 0.41 ^f^	3.7 ± 0.03 ^a^
10	252.64 ± 1.42 ^f^	204.18 ± 2.55 ^l^	59.93 ± 0.39 ^c^	32.97 ± 0.77 ^e^	220.5 ± 0.50 ^a^	6.5 ± 0.06 ^de^	55.9 ± 0.54 ^ac^	7.0 ± 0.04 ^b^
11	937.91 ± 5.85 ^a^	83.55 ± 0.28 ^f^	30.92 ± 0.20 ^a^	1.20 ± 0.07 ^c^	10.3 ± 0.60 ^b^	6.2 ± 0.06 ^g^	54.1 ± 0.49 ^a^	20.1 ± 0.10 ^j^
12	137.25 ± 0.43 ^b^	70.79 ± 0.14 ^e^	51.67 ± 0.18 ^f^	3.73 ± 0.03 ^abc^	55.5 ± 0.50 ^d^	8.5 ± 0.07 ^h^	61.0 ± 0.56 ^d^	39.0 ± 0.22 ^l^
The mean *n* = 12	285.90 ± 1.84	97.50 ± 0.40	51.67 ± 0.22	10.00 ± 0.19	138.7 ± 0.54	6.4 ± 0.05	52.3 ± 0.49	16.6 ± 0.11

^a, b, c, d, e, f, g, h, i, j, k, l^—indicate significant (*α* < 0.05) differences between samples.

**Table 3:** Results of phenolic profiling and ascorbic acid content.

	**TPC** **(mg GEA/g DW)**	**TFC** **(µg QE/g DW)**	**TPAC** **(µg CA/g DW)**	**AA** **(mg AA/g DW)**
	Hydromethanolic extracts			
1	6.19 ± 0.51 ^abc^	402.68 ± 10.46 ^cd^	1679.87 ± 273.39 ^a^	5.16 ± 0.74 ^a^
2	7.27 ± 1.30 ^abcd^	437.70 ± 51.16 ^de^	2167.29 ± 527.83 ^a^	7.29 ± 0.84 ^bc^
3	5.82 ± 0.21^ab^	308.11 ± 33.69 ^abc^	1172.03 ± 55.91 ^a^	5.25 ± 0.10 ^ab^
4	5.83 ± 0.26 ^ab^	265.43 ± 32.50 ^ab^	1068.63 ± 169.50 ^a^	5.12 ± 0.52 ^a^
5	5.69 ± 0.72 ^ab^	207.37 ± 20.47 ^a^	1083.41 ± 134.43 ^a^	5.86 ± 0.57 ^abc^
6	10.13 ± 0.61 ^cd^	619.82 ± 18.88 ^f^	2270.04 ± 276.31 ^a^	7.61 ± 0.04 ^c^
7	11.38 ± 1.11 ^de^	294.63 ± 17.05 ^abc^	2382.53 ± 199.81 ^a^	10.88 ± 0.37 ^d^
8	3.87 ± 0.17 ^a^	230.20 ± 21.23 ^a^	1028.02 ± 43.40 ^a^	5.30 ± 0.52 ^ab^
9	6.89 ± 0.31 ^abc^	386.88 ± 22.48 ^d^	2333.72 ± 21.66 ^a^	7.77 ± 0.59 ^c^
10	9.23 ± 3.07 ^bcd^	432.42 ± 73.97 ^de^	1418.94 ± 136.42 ^a^	5.97 ± 1.92 ^abc^
11	14.32 ± 1.11 ^e^	255.27 ± 34.49 ^a^	4487.74 ± 905.66 ^b^	11.67 ± 0.45 ^d^
12	8.29 ± 0.82 ^bcd^	528.18 ± 20.14 ^ef^	4552.32 ± 749.64 ^b^	6.73 ± 0.40 ^c^
	**TPC** **(mg GEA/g DW)**	**TFC** **(µg QE/g DW)**	**TPAC** **(µg CA/g DW)**	**AA** **(mg AA/g DW)**
	Aqueous extracts			
1	7.66 ± 0.15 ^c^	467.26 ± 5.46 ^d^	2014.18 ± 48.35 ^b^	12.52 ± 0.70 ^ab^
2	6.67 ± 0.12 ^bc^	404.03 ± 23.10 ^c^	2011.45 ± 10.55 ^b^	11.59 ± 0.31^a^
3	5.80 ± 1.16 ^ab^	257.04 ± 7.17 ^ab^	1887.11 ± 26.39 ^b^	10.52 ± 0.44 ^a^
4	5.13 ± 0.26 ^a^	224.56 ± 10.08 ^a^	1219.47 ± 101.83 ^a^	10.78 ± 0.70 ^a^
5	14.28 ± 0.37 ^d^	687.44 ± 31.76 ^e^	3414.55 ± 107.49 ^c^	17.23 ± 0.28 ^c^
6	16.52 ± 0.09 ^e^	773.44 ± 24.31 ^f^	7734.72 ± 169.21 ^g^	14.84 ± 0.39 ^bc^
7	14.53 ± 0.32 ^d^	294.28 ± 2.36 ^b^	6720.06 ± 63.55 ^f^	17.23 ± 0.76 ^cd^
8	4.64 ± 0.29 ^a^	241.30 ± 0.37 ^ab^	1933.87 ± 51.39 ^b^	11.12 ± 0.87 ^a^
9	7.58 ± 0.37 ^c^	437.15 ± 4.44 ^cd^	4354.92 ± 78.61 ^e^	15.55 ± 1.18 ^cd^
10	7.57 ± 0.38 ^c^	430.15 ± 1.24 ^d^	3965.55 ± 79.22 ^d^	17.77 ± 0.66 ^d^
11	5.18 ± 0.20 ^a^	259.57 ± 9.21 ^a^	2129.14 ± 40.41 ^b^	12.17 ± 0.88 ^a^
12	18.26 ± 0.28 ^f^	455.65 ± 22.04 ^c^	4429.79 ± 87.47 ^e^	16.07 ± 0.20 ^cd^

^a, b, c, d, e, f, g^—indicate significant (*α* < 0.05) differences between samples.

**Table 4:** Results of antioxidant activity assays.

	**CUPRAC** **mg AA/g DW**	**ABTS** **mg TE/g DW**	**FRAP** **µmol Fe^2+^/g DW**	**DPPH** **mg TE/g DW**
No	Hydromethanolic extracts			
1	15.13 ± 2.01 ^abc^	64.69 ± 3.31 ^ab^	65.87 ± 3.95 ^ab^	3.06 ± 0.07 ^a^
2	15.56 ± 0.50 ^bc^	64.30 ± 2.62 ^ab^	60.80 ± 4.27 ^ab^	2.97 ± 0.05 ^a^
3	20.67 ± 0.74 ^cde^	60.97 ± 3.57 ^a^	60.04 ± 0.35 ^ab^	3.60 ± 0.04 ^a^
4	20.65 ± 1.97 ^cd^	56.99 ± 6.67 ^a^	61.80 ± 2.99 ^ab^	3.36 ± 0.05 ^a^
5	15.35 ± 2.29 ^bc^	68.28 ± 4.40 ^abc^	61.27 ± 0.02 ^ab^	3.01 ± 0.29 ^a^
6	25.84 ± 1.28 ^ef^	88.61 ± 9.56 ^c^	132.97 ± 9.51 ^d^	7.54 ± 0.27 ^b^
7	31.69 ± 3.16 ^f^	82.58 ± 4.69 ^bc^	111.90 ± 14.12 ^d^	7.85 ± 0.55 ^b^
8	10.42 ± 2.00 ^a^	65.22 ± 6.76 ^ab^	42.47 ± 1.52 ^a^	2.54 ± 0.23 ^a^
9	13.26 ± 0.86 ^ab^	60.61 ± 2.30 ^a^	82.07 ± 5.80 ^b^	3.58 ± 0.61 ^a^
10	24.72 ± 2.53 ^de^	63.49 ± 1.64 ^b^	126.85 ± 13.50 ^d^	2.96 ± 0.99 ^a^
11	46.50 ± 3.09 ^g^	192.82 ± 10.74 ^d^	67.05 ± 8.06 ^ab^	3.84 ± 0.31 ^a^
12	23.23 ± 1.78 ^de^	59.20 ± 3.00 ^a^	107.52 ± 10.78 ^cd^	7.34 ± 0.44 ^b^
	**CUPRAC** **mg AA/g DW**	**ABTS** **mg TE/g DW**	**FRAP** **µmol Fe^2+^/g DW**	**DPPH** **mg TE/g DW**
No	Aqueous extracts			
1	20.38 ± 0.53 ^d^	23.32 ± 0.93 ^d^	139.43 ± 4.21 ^c^	0.84 ± 0.23 ^a^
2	16.29 ± 0.43 ^bc^	22.20 ± 0.89 ^cd^	117.25 ± 4.78 ^bc^	2.13 ± 0.18 ^d^
3	15.68 ± 0.58 ^b^	19.64 ± 0.52 ^bc^	121.64 ± 1.95 ^c^	3.24 ± 0.09 ^e^
4	10.49 ± 0.24 ^a^	23.94 ± 0.65 ^d^	90.45 ± 6.19 ^a^	1.38 ± 0.26 ^bc^
5	28.84 ± 1.13 ^e^	39.47 ± 0.82 ^f^	224.55 ± 13.27 ^e^	0.80 ± 0.05 ^a^
6	40.39 ± 0.54 ^g^	41.66 ± 0.22 ^f^	317.83 ± 10.18 ^g^	1.80 ± 0.11 ^a^
7	36.75 ± 1.35 ^f^	49.04 ± 1.34 ^g^	287.46 ± 5.36 ^f^	1.90 ± 0.11 ^d^
8	12.39 ± 0.17 ^a^	15.50 ± 0.77 ^a^	87.87 ± 7.32 ^a^	1.01 ± 0.05 ^ab^
9	18.40 ± 0.60 ^cd^	19.05 ± 0.48 ^b^	179.82 ± 6.66 ^d^	2.95 ± 0.13 ^e^
10	19.82 ± 0.58 ^d^	24.06 ± 0.38 ^d^	142.05 ± 3.85 ^c^	2.03 ± 0.12 ^d^
11	12.96 ± 0.79 ^a^	30.60 ± 0.64 ^e^	95.95 ± 3.96 ^a^	1.17 ± 0.14 ^ab^
12	20.15 ± 0.62 ^d^	28.11 ± 1.29 ^e^	181.41 ± 7.12 ^d^	1.83 ± 0.08 ^cd^

^a, b, c, d, e, f, g^—indicate significant (*α* < 0.05) differences between samples.

## Data Availability

The original contributions presented in this study are included in the article. Further inquiries can be directed to the corresponding author.

## Abbreviations

The following abbreviations are used in this manuscript:

HPLC	High-Performance Liquid Chromatography
FAAS	Flame Atomic Absorption Spectrometry
FRAP	Ferric-Reducing Antioxidant Power
CUPRAC	Cupric-Reducing Antioxidant Capacity
DPPH	2,2-diphenyl-1-picryl-hydrazyl-hydrate
ABTS	Radical-Scavenging Activity
DW	Dry Weight
